# Leveraging policy to reduce chronic opioid use by educating and empowering community dwelling adults: a study protocol for the TAPERING randomized controlled trial

**DOI:** 10.1186/s13063-019-3508-z

**Published:** 2019-07-09

**Authors:** Justin P. Turner, Patricia Caetano, Cara Tannenbaum

**Affiliations:** 1 Faculté de Pharmacie, Université de Montréal, Montréal, QC Canada; 2 Centre de Recherche de l’Institut Universitaire de Gériatrie de Montréal, Montréal, QC Canada; 3 Faculty of Health Sciences, University of Manitoba, Winnipeg, MB Canada; 4 Provincial Drug Programs, Government of Manitoba, Winnipeg, MB Canada; 5 Michel Saucier Endowed Chair in Pharmacology, Health and Aging, Facultés de Médecine et de Pharmacie, Université de Montréal, Montréal, QC Canada

**Keywords:** Analgesics, Opioids, Deprescribing, Patient education, Government, Policy, Chronic pain

## Abstract

**Background:**

Opioid use has risen to epidemic proportions across Canada, with increasing evidence of harms including accidental overdose and death. Policy-makers have called for effective approaches to promote opioid reduction. One promising method from deprescribing randomized trials is to empower patients through direct-to-patient education. The current trial will evaluate the effectiveness of a government-led mail-out of educational information to adult community-dwelling, chronic opioid users on the reduction of opioids compared to usual care.

**Methods:**

This is a pragmatic, prospective, cluster randomized, parallel-arm controlled trial, comparing mailed distribution of a direct-to-patient educational brochure for chronic opioid use (intervention arm) to usual care (control arm). Eligible participants from across Manitoba, Canada, will be identified by the Provincial Drug Programs Branch within the Manitoba Health, Seniors and Active Living Department of the Manitoba Government, allocated to primary care providers, and the latter will be randomized in clusters of family medicine practices to achieve a 1:1 ratio. The primary outcome is complete cessation of opioids after 6 months assessed using Drug Program Information Network data. Secondary outcomes include ≥ 25% dose reduction in the mean morphine milligram equivalent (MME) daily dose, reduction of daily dose to < 90 mg MME, or therapeutic switch to another opioid or non-opioid medication. Data will be analyzed using intent-to-treat generalized estimating equations.

**Discussion:**

This trial will test the efficacy of a population-based, wide-scale, government-led direct-to-patient educational initiative to drive reductions in chronic opioid use by community-dwelling adults across Manitoba.

**Trial Registration:**

ClinicalTrials.gov, ID: NCT03400384. Registered on 18 January 2018.

**Electronic supplementary material:**

The online version of this article (10.1186/s13063-019-3508-z) contains supplementary material, which is available to authorized users.

## Background

Opioids are frequently prescribed for acute pain, palliative care and cancer pain, supported by evidence from 12-week randomized controlled trials [1, 2]. However, the use of opioids has grown steadily in the last two decades despite limited evidence of their long-term benefit in non-cancer patients [3]. When used chronically, opioids have been linked in a dose-response and duration of use fashion with fractures [4–6], myocardial infarction [7, 8], sexual dysfunction in men [9], and motor vehicle accidents [10].

In parallel to their increased use, opioid-induced harm and related deaths have tripled in Canada over the past decade, leading the government to declare the opioid crisis a federal priority [11–13]. In 2016, approximately 13% of the Canadian adult population consumed an opioid medication [14], with 16 Canadians hospitalized each day due to accidental opioid overdose [12]. Opioid-related deaths exceeded motor vehicle accidents fatalities by 34%, leading to over 2500 Canadians dying as a direct result of opioids [15, 16]. In the province of Manitoba, approximately 11 people per 100,000 were hospitalized in 2016 as a result of opioid toxicity [11], and approximately nine people per 100,000 died in 2017 as a result of consuming opioids [17]. While some of these deaths are due to illicit opioid use, approximately one quarter of hospitalizations in Canadians aged 65 years and over occurred when opioids were taken as prescribed [11]. Furthermore, one third of opioid-related deaths in the province of Ontario occurred in people with an active opioid prescription [18].

There is limited research demonstrating practical and effective interventions to reduce opioid use, leaving policy-makers ill-informed about how to promote fewer prescriptions. Findings can only be extrapolated from jurisdiction-wide initiatives to curb other inappropriate drugs such as benzodiazepines. Introduction of triplicate prescription pads in the state of New York produced a 33% reduction in benzodiazepine use [19]. Similarly, prescription monitoring systems for opioids have recently been implemented in North America producing a 10% reduction in prescription opioid use in Medicaid enrollees [20, 21]. Rescheduling alprazolam to restrict access in Australia yielded a decrease in alprazolam use, but an increase in benzodiazepine substitutions and overdose deaths [22, 23]. This pattern was replicated with the removal of OxyContin® from formularies across North America, resulting in a switch to hydromorphone and heroin [21], followed by increased heroin-related deaths [24]. These observations suggest that while monitoring prescribing and restricting the supply of prescription opioids may be one way to reduce use, additional efforts are required to educate patients about the hazards of the drug class as a whole so that equally harmful substitutions do not occur.

A couple of initiatives highlight the potential for policy-makers to reduce inappropriate medication use through widespread education. In South Australia, a multidisciplinary regional educational campaign to healthcare providers and patients to reduce sedative-hypnotics showed promise, resulting in a 19% reduction in benzodiazepines sustained over 24 months [25]. Direct patient education also successfully diminished benzodiazepine prescriptions in Quebec, Canada, during the EMPOWER randomized trial, where community-dwelling chronic benzodiazepine users received a direct-to-patient education brochure by mail, describing the harms of sedative-hypnotics [26]. Sixty-two percent of trial participants initiated conversations with a healthcare provider and 27% discontinued their benzodiazepine prescription within 6 months [26]. Although these projects produced significant reductions in sedative-hypnotic use, important questions remain unanswered for opioids: (1) Will the intervention have the same magnitude of effect for chronically prescribed opioids?; and (2) Are the results externally reproducible at a population level? In 2017 a policy-research collaboration was struck between the Canadian Deprescribing Network [27] and the Government of the province of Manitoba to try to answer these questions.

## Objectives

The objective of the trial is to evaluate the effectiveness of a government-led mail-out of educational information directly to adult, community-dwelling, chronic opioid consumers on the reduction of opioid utilization, compared to usual care, as measured by the cessation or dose reduction of opioids after 6 months post intervention. The acronym TAPERING stands for “*T*rial *A*pplying *P*olicy to *E*liminate or *R*educe *I*nappropriate *N*arcotics in the *G*eneral-population.”

## Methods

### Trial design

This Manitoba Health, Seniors and Active Living policy initiative is being implemented across the provincial health jurisdiction of Manitoba, Canada. This is a pragmatic, prospective, cluster randomized, parallel-arm controlled trial, comparing dispatch of a direct-to-patient educational brochure in the mail (intervention arm) to usual care (control arm). A cluster design was chosen to prevent contamination between the intervention and control arms amongst patients who attended the same family medicine clinics (each family medicine clinic forms a cluster unit). By randomizing individuals according to practice unit, the cluster design reduces the potential for bias if a physician has patients from both the intervention and control arms, as the intervention group receives a letter and direct-to-patient educational brochure with instructions to talk to their family physician to reduce their opioid use. The main outcome is at the level of the individual patient. The pragmatic approach allows participants to react naturally to the educational material in the context of a real-life setting. The control group is in fact wait-listed to receive the educational material after completion of the 6-month trial.

## Participants

### Trial setting

The trial is being conducted across the province of Manitoba, Canada. Manitoba is one of 13 healthcare jurisdictions in Canada, that serves a total population of approximately 1.4 million. The policy partner is the Provincial Drug Programs Branch within the Manitoba Health, Seniors and Active Living Department of the Government of Manitoba. This department strives to meet the health needs of individuals, families and their communities by leading a sustainable, publicly administered health system that promotes well-being and provides the right care, in the right place, at the right time. In line with this vision, the Provincial Drug Programs Branch is committed to implementing evidence-based approaches to reduce the use and harms associated with inappropriate medication use. The Branch has access to all administrative prescription medication claim data for the entire population through the Drug Program Information Network. The network collects information on all medications dispensed from community-pharmacies within Manitoba, regardless of insurance coverage or final payer.

### Inclusion criteria

All community-dwelling adults (aged 18 years and over) who are registered to receive healthcare benefits in Manitoba are eligible for inclusion if they meet the following criteria: chronic opioid use, defined as receiving ≥ 90 days’ supply of opioids during the past 120 days. The supply of opioids is calculated as the sum of all prescriptions dispensed within Manitoba for the following opioids: fentanyl, hydromorphone, meperidine (pethidine), morphine and oxycodone, as per International Non-proprietary Names and Anatomical Therapeutic Chemical (ATC) codes recommended by the World Health Organization [28], and Drug Identification Numbers (DIN) according to Health Canada. Fentanyl, hydromorphone, morphine and oxycodone are considered strong opioids usually prescribed for chronic pain, unlike codeine and tramadol [14]. Therefore, tramadol and codeine will be excluded from the intervention, yet included in the analysis to determine if substitution occurs. In Manitoba, the number of days of medication supplied must be submitted by the pharmacist at the time of dispensing. Additionally, all opioids, including low-dose codeine preparations, are entered into the Drug Program Information Network such that all authorized prescribers can access patient medication histories to check for appropriateness, existing or past treatments, and potential drug interactions.

### Exclusion criteria

The Drug Program Information Network (DPIN) data does not contain indications for use; therefore, the following exclusion criteria will be used to try to exclude patients with cancer: (1) receiving palliative care (defined as receiving medications from the Palliative Care Drug Access Program), (2) receiving treatment for cancer within the previous 12 months (defined as receiving medications from the Home Cancer Drug Program). Participants will also be excluded if they meet one of the following criteria: (1) are residing in a nursing home, (2) are receiving opioids from within a hospital setting exclusively, or (3) have been diagnosed with dementia (defined as receiving memantine or a cholinesterase inhibitor in the previous 12 months).

### Recruitment and allocation

**Fig. 1:**
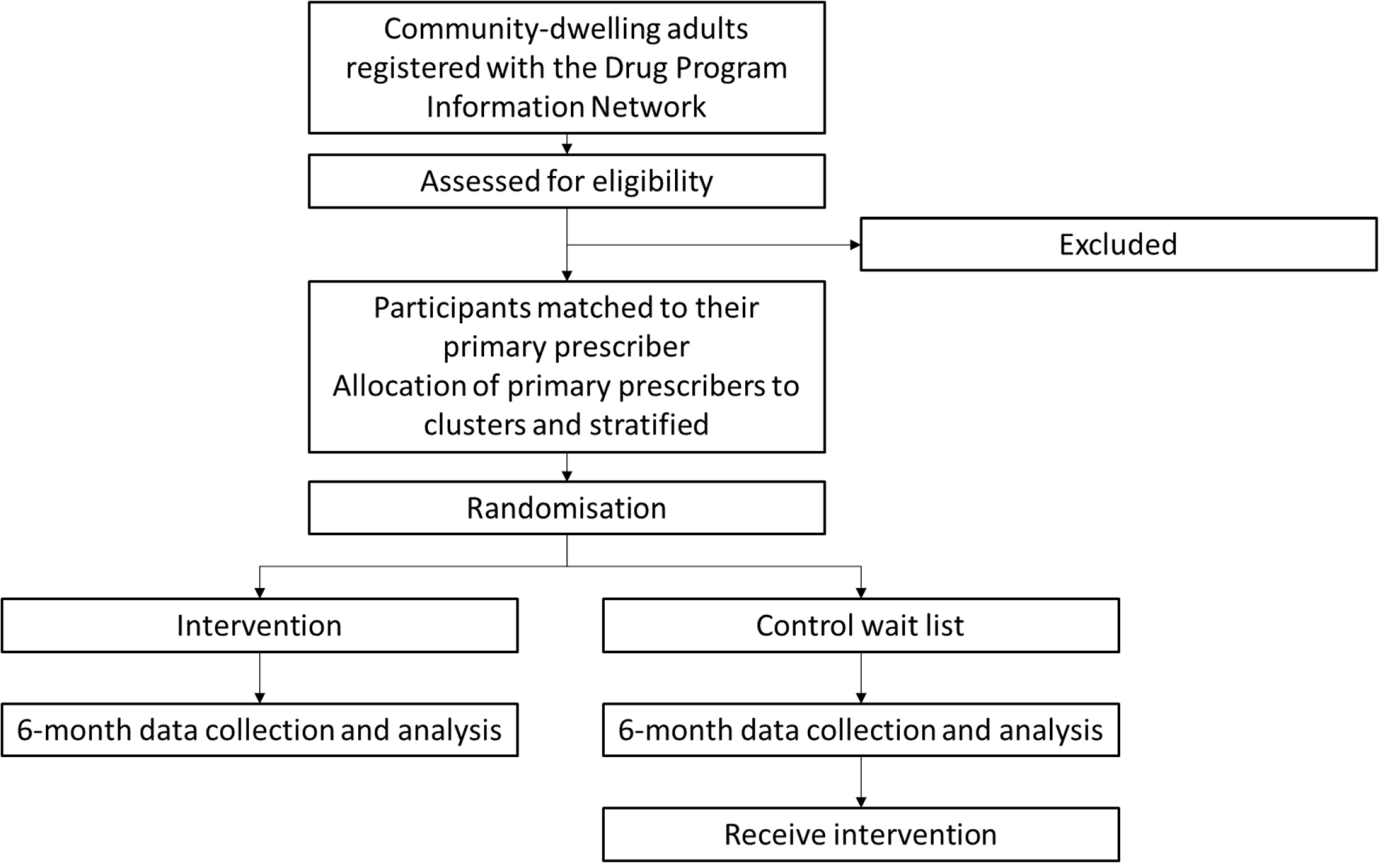
A flow chart for the study process

**Fig. 2:**
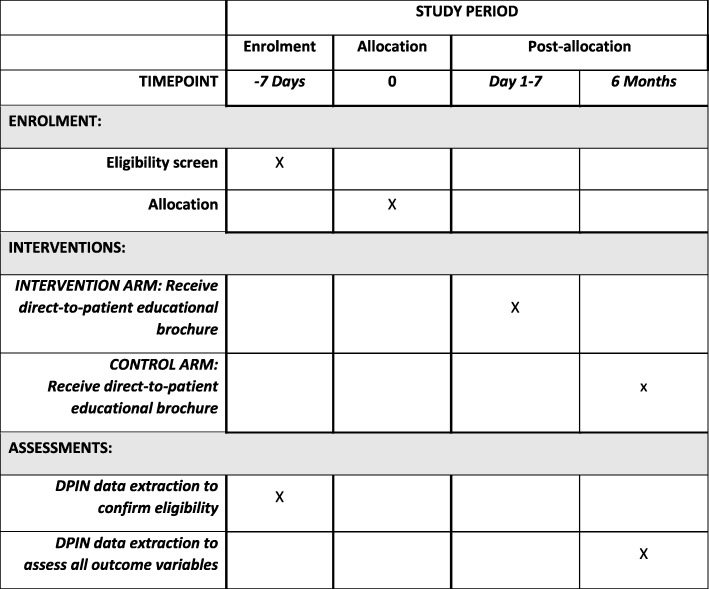
Schedule of enrollment, intervention and assessment

Recruitment will occur via the Manitoba Provincial Drug Programs Branch, which monitors all individuals registered in the Drug Program Information Network (DPIN) receiving opioids and meeting eligibility criteria. The DPIN contains information for all prescription medications dispensed by community pharmacies in Manitoba for all residents of Manitoba. Eligible participants will be identified according to prescription claims in the DPIN. The primary prescriber for each participant will be deemed to be the prescriber who has prescribed the most medications for the patient in the past 6 months according to DPIN data. Clusters will be defined as geographically co-located family medicine clinics, with physicians allocated to clusters according to the postal code of their registered practicing address. Participants will be allocated to the cluster according to their primary prescriber. Clusters (and the participants therein) will be stratified and randomized to one of two arms, intervention or control in a 1:1 ratio. Six cluster strata will be created using a computer algorithm: five strata according to the number of physicians registered at an individual six-digit postal code (1–10, 11–20, 21–30, 31–40, > 40), and a sixth stratum for prescribers with no identifiable practicing address. A 1:1 allocation ratio will be assigned by an independent statistician using a random number sequence generator across all six strata in block sizes of two (Fig. 1). Allocation will be concealed from both individuals and clusters. Figure 2 details the Standard Protocol Items: Recommendations for Interventional Trials (SPIRIT) schedule of enrollment, interventions and assessment used within this trial. The study protocol was developed in accordance with SPIRIT 2013 (Additional file 1) [29].

Following the randomization process, cover letters and educational brochures will be mailed to participants in the intervention group from the Provincial Drugs Branch mail centre over the course of 1 week. This will allow a practical workload for the mail center and reduce the risk of family medicine clinics being inundated with questions from patients all in 1 day.

It will be impossible to blind participants and their health care providers to the intervention assignment because participants in the intervention arm will receive the educational brochure and are encouraged to discuss it with their healthcare provider. Additionally, the College of Physicians and Surgeons of Manitoba will alert their members to a public awareness campaign relating to opioids for the treatment of chronic non-cancer pain via their newsletter. Despite the inability to blind participants, the analyses will be carried out by a statistician who is blinded to the allocation.

### Intervention

An opioid educational brochure will be dispatched by mail to the participants in the intervention group. The 20-page direct-to-patient educational brochure was created using social constructivist learning theory and self-efficacy theory, in a similar format to the brochures that were developed for the EMPOWER and D-PRESCRIBE trials [26, 30, 31]. The evidence-based brochures were iteratively reviewed by healthcare professionals, Indigenous people, and focus groups of patients who used opioids chronically for the treatment of chronic non-cancer pain. The brochure was produced and reviewed in both of Canada’s official languages, English and French.

The brochure includes: the name of each dispensed opioid medication and an explanation of the difference between chronic and acute pain; questions and infographics designed to elicit cognitive dissonance about the potential harms of opioid use and the risk of hospitalization or death; self-assessment for tolerance or side effects; a list of alternatives to opioids for chronic non-cancer pain, including details on self-management strategies such as pacing and positive thinking; a peer champion story to augment self-efficacy; information about tapering opioids, including a link to an online calculator for dose reduction (www.deprescribingnetwork.ca/tapering) and a list of local resources to consult. The brochure advises patients at multiple points to consult a healthcare professional prior to making any changes to their opioid consumption, and provides warnings about the risks of acute withdrawal. The brochure uses a size 14-font and eighth-grade reading level. In line with federal government requirements, the educational intervention also includes a sex and gender-based analysis lens, informing readers of sex-specific risks of opioid-related adverse events and associations between opioids and sex hormones. Indigenous representatives ensured the brochure was culturally sensitive. In an attempt to increase patient engagement with the educational brochure, each brochure will be tailored to reflect the specific opioid that the patient received in the previous 120 days. For participants prescribed a single opioid molecule, e.g., morphine, the cover page of the brochure will state “You are taking the following opioid medication: morphine”. Participants who received multiple opioids during the 120 days prior to the trial will be posted a generic brochure that states “You have been taking one or more of the following opioids medications: …” and includes the names of all opioids available in Manitoba. The content of all the brochures will be otherwise identical, with page 2 listing all available opioids in Manitoba to ensure that participants can identify if they are prescribed a substitute opioid.

Participants in the intervention group will receive a cover letter in the envelope along with the educational brochure. The cover letter will explain why the recipient is receiving the brochure and that the brochure provides general information. The letter will assure recipients that their right to both access and financial coverage of medications will not be affected. Additionally, the cover letter will highlight that the brochure specifically focuses on chronic non-cancer pain, to specify the intended target audience. Finally, the letter will also advise recipients to discuss their individual medical treatment with their healthcare provider.

Prescribers will not be directly targeted by the intervention; however, there will be supports in place to assist them with opioid deprescribing. First, an online opioid reduction calculator was created to provide support for a 10–20% dose reduction for opioids over a 1–4 week interval. The calculator produces a printable report that includes the dose, frequency of medication administration, date of dose reductions and a graphical representation of the opioid prescribed to assist both the prescriber and patient in the dose reduction process (available at www.DeprescribingNetwork.ca/tapering). The web address will be included in the educational brochure, along with instructions to talk to a physician or pharmacist. Additionally, the College of Physicians and Surgeons of Manitoba (CPSM) provides access to opioid prescribing support services for all prescribers across Manitoba. CPSM and one of the opioid prescribing support physicians reviewed and approved the intervention materials and were aware of the mail-out dates.

## Outcome measures

### Primary outcome measure

The primary outcome is complete cessation of opioids after 6 months, assessed at the patient level using the Drug Program Information Network (DPIN) data within the Provincial Drugs Program Branch. Deprescribing will be defined as no opioid dispensed or on hand in the 60 days following the 6-month follow-up period.

### Secondary outcome measures

Secondary outcomes include dose reduction and/or therapeutic switch. Dose reduction will be defined in two ways. First, we will consider dose reduction as being a ≥ 25% decrease in the mean daily morphine milligram equivalent (MME) dose comparing the month immediately prior to the intervention with the month after the 6-month follow-up period. Additionally, we will analyze any dose reduction and absolute dose reduction (in daily MMEs) for the same time period. Additionally, in accordance with the new Canadian Guideline for Opioid Therapy and Chronic Non-Cancer Pain [32], we will investigate the proportion of patients who were receiving a monthly average of ≥ 90 mg MME/day in the month immediately prior to the intervention who reduce their dose to an average of < 90 mg MME/day in the seventh month. New dispensing of a prescription for a lower strength, or supply of a reduced quantity of tablets/patches will be considered in the dose reduction calculation. Where substitution to an alternative opioid occurs, opioid doses will be converted to daily MME to determine dose reduction. The MME for all opioids on hand will be calculated for the aforementioned periods of time and then used to determine the mean daily MME. It is possible that other medications may be initiated in an attempt to reduce opioid dose and maintain pain control. For example, gabapentinoid use is increasing in the United States as a potential response to the opioid crisis [33]. A therapeutic switch will be indicated by a new dispensing for an alternative medication class. New dispensing will be defined as dispensing a medication that has not been dispensed or on hand from prior dispensing in the 12 months prior to the initial dispatch of patient educational brochures. Subgroup analysis will consider the impact of participants’ sex and age in primary and secondary outcomes.

### Sample size

This is a population-level trial with no intention to recruit a minimum predetermined sample size. However, to ensure feasibility of the trial, the minimum sample size required to identify the primary outcome of complete cessation of opioids at 6 months was considered. The sample size calculation to detect a minimum 5% difference in cessation of opioids between the intervention arm compared to the control arm with a power of 90% and an α level of 0.05 (two-sided) is a total of 1044 (522 per arm). This assumes there will be a 5% absolute cessation rate in the control arm, and a minimum 10% absolute cessation rate of opioids in the intervention arm. Consideration must be given to the cluster design of the trial [34]. Assuming a mean of five eligible participants per physician, and a conservative intracluster correlation of 0.2 based on previous research in primary care [35], we increased the sample size by a factor of 1.8 to 1880 (940 per arm). The intervention (direct-to-patient educational brochure) will be mailed to all adults in Manitoba who meet the inclusion and exclusion criteria. Participants allocated to the intervention arm will have theirs dispatched at baseline, while participants in the control arm will have theirs dispatched 6 months later. Preliminary analysis identified that over 5000 people in Manitoba were prescribed opioids chronically in accordance with the inclusion and exclusion criteria, thus the proposed sample size is feasible.

## Measurement, definitions and data collection

Data will be collected from the Drug Program Information Network and used to identify persons who meet the inclusion criteria. Data will be extracted at 6 months following the intervention to measure primary and secondary outcomes. Extracted data will include patient age, sex and pharmacy prescription records. Data will also be extracted at the level of the prescriber, including six-digit postal code, number of patients for whom opioids are prescribed, dose of opioids prescribed (which will be converted to MME) and number of years since registration. All opioid medications will be coded as International Non-proprietary Names and Anatomical Therapeutic Chemical (ATC) codes (NO2A) recommended by the World Health Organization [28], and Drug Identification Numbers (DIN) according to Health Canada to enable detection of dose (available here https://health-products.canada.ca/dpd-bdpp/index-eng.jsp). Substitutions for the target medications will be defined as pharmacy claims for the following medication classes where they have not been claimed in the previous 12 months: other opioids including codeine and tramadol (N02A), acetaminophen (N02BE), non-steroidal anti-inflammatory drugs (NSAIDs) (oral use (M01A) and topical use (M02AA)), gabapentin (N03AX12), pregabalin (N03AX16), low-dose tricyclic antidepressants (N06AA), duloxetine (N06AX21), carbamazepine (N303AF01) and disease-modifying anti-rheumatic drugs (DMARDs) (aminosalicylic acid and similar agents (A07EC), folic acid analogs (L01BA), selective immunosuppressants (L04AA), other immunosuppressants (L04AX), specific antirheumatic agents (M01C), aminoquinolines (P01BA)).

### Data management

Only authorized personnel from within the Provincial Drugs Program Branch will have access to patient or healthcare- provider-level data consistent with provincial government oversight roles and responsibilities and in accordance with relevant provincial privacy legislation. A link between Drug Program Information Network data and patient identifying data will be created solely for the purpose of the mail out. Data extracted by the Provincial Drugs Program Branch will be anonymized to ensure removal of patient and healthcare provider identifying material. Anonymized data will be stored in a secure folder and used for all analyses.

### Statistical methods

Differences between baseline characteristics of the intervention and control arms will be analyzed using descriptive statistics, with chi-square tests for categorical data and *T* tests to compare the means of normally distributed variables.

The main outcome, cessation of opioid prescriptions, will be analyzed using an intention-to-treat approach. The unadjusted risk differences (with 95% confidence intervals) will be calculated via generalized estimating equations with discontinuation as a binary outcome, assessed for each patient at 6 months post intervention and an identity link, using patients as the unit of analysis. The same method will be applied for secondary outcomes. A *p* value of < 0.05 will be considered as statistically significant.

It will not be possible for participants to withdraw from the trial. Standard DPIN protocol will be employed to identify participants who leave the province of Manitoba or die during the follow-up period.

## Discussion

Opioid use can result in high personal costs to the individual and society through adverse drug events, hospitalization and death [36]. Opioid use has increased dramatically across North America in the past decade. Implementation of policies to reduce opioid-related harms is being driven by heightened public awareness, frightening overdose statistics and a changing political landscape [12, 21, 37]. Although several healthcare jurisdictions in Canada have implemented policies in an attempt to reduce opioid use, their effect remains undetermined.

The strengths of this trial include the large, representative sample that includes all people in Manitoba regardless of their insurance coverage, thus increasing external validity. Likewise, internal validity is strengthened through randomization of all eligible participants. Clustering at the level of the family medicine clinic reduces the chance of bias introduced through hallway conversations where either participants or physicians discuss the intervention when they pass each other within the clinic. Stratification according to the size of the family medicine clinic produces a number of strengths. It permits the intervention to be delivered evenly across different community settings in Manitoba, increasing its external generalizability. Furthermore, the structures, systems and processes within small and large clinics may be different, thus providing diverse opportunities such as the availability of an individual physician or potential for conversations between colleagues. The pragmatic approach to the trial is another strength as it ensures the trial reflects real-world settings.

The trial has some limitations. First, neither the indication for use of a medication (opioids and therapeutic switches) nor clinical appropriateness is available within the DPIN data. The definition of chronic non-cancer pain relies on excluding patients on the basis of information contained within datasets, including treatment for cancer or palliative care, or receipt of opioid therapy solely through the hospital system. These assumptions may not correctly identify all patients with chronic non-cancer pain; however, this limitation applies equally to both arms, thus restricting its impact on the results. Second, the algorithms to identify eligible participants may be imperfect, thus resulting in ineligible participants being sent the intervention, or eligible participants not receiving the intervention. Similarly, due to the randomization process, we expect this limitation to apply equally to both arms. Third, the exclusion of codeine and tramadol as included opioids may cause participants and/or physicians to consider these as safer alternatives resulting in an increase in their prevalence. This switch will be monitored as one of the secondary outcomes; however, we suspect that recent pharmacist education to reduce over-the-counter codeine use may moderate the switch to codeine. Fourth, it is impossible to measure if participants switch to non-dispensed opioids (i.e., illegal opioid use). Fifth, in November 2016 the Federal Government of Canada convened a “National Conference on Opioids” then in May 2017 the updated Canadian guideline on opioid therapy and chronic non-cancer pain was released [32]. Therefore, the debate on opioid use has been raised at the public and healthcare professional level, meaning that many individuals have already discontinued their opioids, resulting in a potentially biased sample at baseline. Similarly, there may be a larger than expected discontinuation rate in the control arm due to continued media coverage of the opioid crisis and other interventions to reduce opioid use.

This trial employs a novel approach to opioid reduction by directly engaging community-dwelling chronic opioid users to initiate conversations with their healthcare providers about their opioid use and alternate treatments for pain. This approach has successfully led to the reduction of inappropriate sedative-hypnotic and NSAID prescriptions within the boundaries of well-designed and structured randomized controlled trials [26, 31]. To our knowledge, this is the first government-led, population-level, pragmatic, prospective, cluster randomized, parallel-arm controlled trial of targeted direct-to-patient education to reduce chronic opioid consumption in the general population. The trial results will help inform future jurisdiction-level initiatives to reduce opioid-related harms in the community setting.

## Additional file

Additional file 1: Standard Protocol Items: Recommendations for Interventional Trials (SPIRIT) 2013 Checklist: recommended items to address in a clinical trial protocol and related documents* (DOC 123 kb)

## Abbreviations

ATC: Anatomical Therapeutic Chemical
DIN: Drug Identification Numbers
DMARDs: Disease Modifying Anti-Rheumatic Drugs
DPIN: Drug Program Information Network
MME: Morphine milligram equivalents
NSAIDs: Non-steroidal anti-inflammatory drugs
TAPERING: *T*rial *A*pplying *P*olicy to *E*liminate or *R*educe *I*nappropriate *N*arcotics in the *G*eneral-population
